# Molecular diversity of Pseudoscorpiones in southern High Appalachian leaf litter

**DOI:** 10.3897/BDJ.12.e115928

**Published:** 2024-01-11

**Authors:** Ernesto Recuero, Michael S. Caterino

**Affiliations:** 1 Clemson University, Clemson, United States of America Clemson University Clemson United States of America

**Keywords:** soil diversity, megabarcoding, species delimitation, Asheville Depression, Arachnida, Appalachia

## Abstract

The Pseudoscorpiones fauna of North America is diverse, but in regions like the southern Appalachian Mountains, they are still poorly documented with respect to their species diversity, distributions and ecology. Several families have been reported from these mountains and neighbouring areas. Here we analyse barcoding data of 136 specimens collected in leaf litter, most of them from high-elevation coniferous forest. We used ASAP as a species delimitation method to obtain an estimation of the number of species present in the region. For this and based on interspecific genetic distance values previously reported in Pseudoscorpions, we considered three different genetic Kimura two-parameter distance thresholds (3%/5%/8%), to produce more or less conservative estimates. These distance thresholds resulted in 64/47/27 distinct potential species representing the families Chthoniidae (33/22/12 species) and Neobisiidae (31/25/15) and at least six different genera within them. The diversity pattern seems to be affected by the Asheville Depression, a major biogeographic barrier in this area, with a higher diversity to the west of this geographic feature, particularly within the family Neobisiidae. The absence of representatives from other families amongst our studied samples may be explained by differences in their ecological requirements and occupation of different microhabitats.

## Introduction

The order Pseudoscorpiones, commonly known as false scorpions or book scorpions, are a group of small arachnids, characterised by their pedipalps ending in chelae, resembling true scorpions, but lacking the elongate metasoma ending in a sting. They go through three post-embryonic stages, protonymph, deutonymph and tritonymph, before they become adults (Weygoldt 1969). They are an old group, with fossils dating back to the Devonian (Shear et al. 1989) and moderately diverse. Currently, 4177 extant species are recognised in the world, including 25 families and 471 genera (World Pseudoscorpiones Catalog 2022). They are predators feeding on different kinds of small organisms, particularly other arthropods and they can be found in a broad array of habitats, from caves to intertidal environments, frequently being abundant in leaf litter communities (Weygoldt 1969, Muchmore 1973). Despite their small size and terrestrial habits, some species are able to effectively disperse over long distances thanks to their phoretic behaviour, in which they attach themselves to other organisms, frequently flying ones, to colonise other areas (Beier 1948).

Pseudoscorpions are diverse in North America, with 432 species known from the USA, 173 from Mexico and 26 from Canada (World Pseudoscorpiones Catalog 2022). Most American species were described during the 20^th^ century by specialists such as C. C. Hoff, J. C. Chamberlin and, particularly, W. B. Muchmore, who described over one third of the species known from North America. The taxonomic research on American pseudoscorpions has been declining during the 21^st^ century, but new species are still found and described regularly, as recently as in 2020 (Harvey and Cullen 2020, Sammet et al. 2020). The diversity of the group in the southern Appalachians is still unclear. Lawson (1967) reported 17 species, including one potentially undescribed taxon. A more recent, unpublished list, compiled by Muchmore and Cokendolpher (available at: https://www.discoverlife.org/nh/cl/GSMNP/arachnid/pseudoscorpion/#Overview), mentions up to 51 species with confirmed or potential presence in the Great Smoky Mountains National Park and other areas of the southern Appalachians, including representatives of eight families, most belonging to the Chthoniidae Daday, 1889 (21 spp), Neobisiidae Chamberlin, 1930 (11 spp) and Chernetidae Menge, 1855 (10 spp).

In the current paper, we present the results for Pseudoscorpiones from a broad molecular barcoding project aimed at characterising the arthropods occurring in leaf litter of the higher elevations of the southern Appalachian Mountains. Our results, depicting the genetic diversity of litter pseudoscorpions mostly from southern Appalachian sky-islands, formed by relict red spruce and Fraser fir forests, provides a novel view of the diversity and distribution in the southern Appalachians of these small animals.

## Material and methods

**Figure 1.**
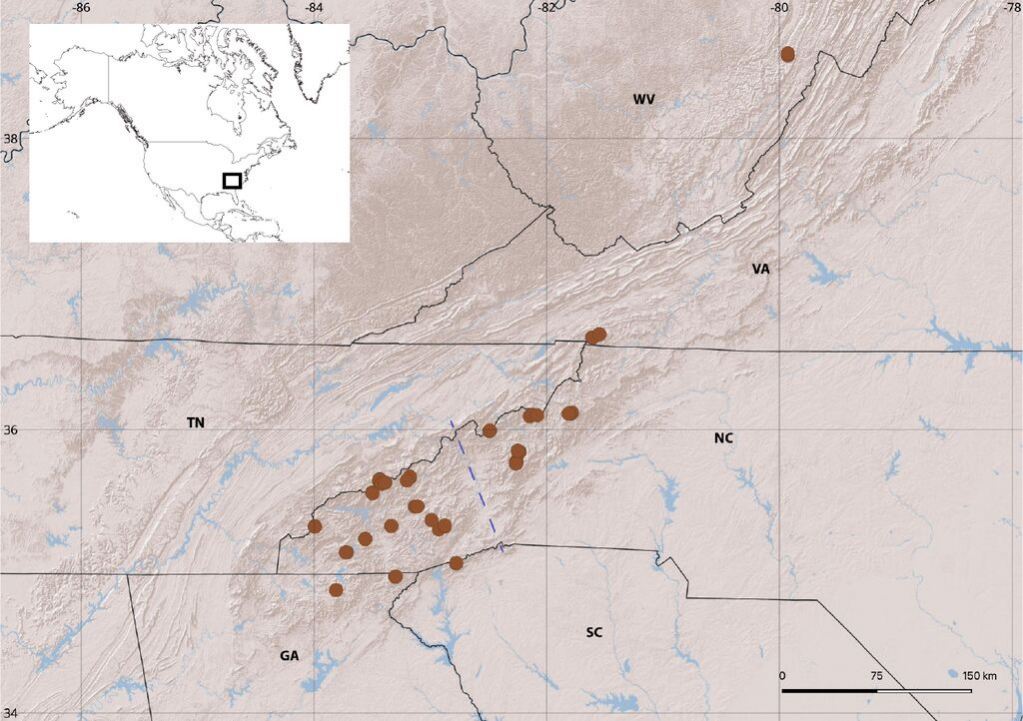
Localities of barcoded Pseudoscorpiones in the southern Appalachian Mountains. Dotted line indicates the position of the Asheville Depression barrier. The inset shows the location of the region of study.

A detailed description of sampling methodology and data generation is provided in Caterino and Recuero (2023a). Pseudoscorpion specimens were collected from leaf litter samples sifted with 8 mm mesh sifters and processed in Berlese funnels. Sampled localities (Fig. 1, Suppl. material 1) included high-elevation coniferous spruce-fir forest, but also lower elevation forests with typically broadleaf deciduous and evergreen litters, in most cases visited once in spring and once in autumn. Samples were fixed in 100% ethanol. Specimens from each locality and each collecting event were sorted to morphospecies, based on their general phenotypic similarity. One specimen per morphospecies, locality and collecting event was photographed by focus stacking (https://www.flickr.com/photos/183480085@N02/albums/72157710331403331) and then prepared for sequencing. Specimens were digested in Proteinase K and DNA extracted with the Mag-Bind HDQ Blood and Tissue kit (Omega BioTek) on a Hamilton Microlab Star automated liquid handling system. Before the extraction, digested vouchers were recovered, preserved in 95% ethanol with a few drops of 100% propylene glycol and housed at the Clemson University Arthropod Collection (CUAC; Suppl. material 1). We amplified a 421 bp Cytochrome *c* oxidase subunit I (*COI*) minibarcode fragment using PCR and primer pair BF2-BR2 (Elbrecht and Leese 2017). Primers were tagged with 9 bp indexes to generate individual combinations and allow multiplexed sequencing (Meier et al. 2015), on either Nanopore or Illumina platforms following their specific protocols. Sequences are deposited in GenBank (Suppl. material 1).

We used PAUP* v.4.0a (Swofford 2003) to build a neighbour-joining tree, based on Kimura 2-parameter distances (Kimura 1980) reflecting the genetic distances between sequenced specimens (Suppl. material 2), using a spider (*Ceratinella* Emerton, 1882) and a scorpion (*Alpiscorpius* Gantenbein, Fet, Largiader & Scholl, 1999) (Graham et al. 2012, Caterino and Recuero 2023a) as outgroups. Additionally, we performed single locus, genetic distance-based species delimitation analyses to generate preliminary hypotheses regarding the number of species present in the studied populations. Specifically, we used ASAP (Assemble Species by Automatic Partitioning; Puillandre et al. (2020)), generating different delimitation partitions with different distance thresholds. For this analysis, we also implemented Kimura 2-parameter distance correction. Genus level identifications of many of the sequenced specimens were achieved by aligning our barcodes with all sequences available from GenBank (Suppl. material 3, searched 1 November 2023) and determining their phylogenetic relationships. This data matrix was analysed with Maximum Likelihood methods using W-IQ-Tree (Trifinopoulos et al. 2016, available at http://iqtree.cibiv.univie.ac.at), with automated substitution model selection (GTR+F+I+G4 chosen under the Bayesian Information Criterion) and 1000 ultrafast boot­strap replicates to measure branch support of the obtained phylogenetic reconstruction.

## Data resources

The data resources associated with this paper comprise an Excel file (Suppl. material 1) with associated voucher data, unique identifiers and GenBank codes and a nexus alignment file including all new barcode sequences (Suppl. material 2).

## Results

Our sampling included 186 extracted specimens, 137 of which were successfully sequenced (Suppl. material 1) and, amongst the latter, we could recover 125 vouchers for eventual morphological study (Suppl. material 2).

**Figure 2.**
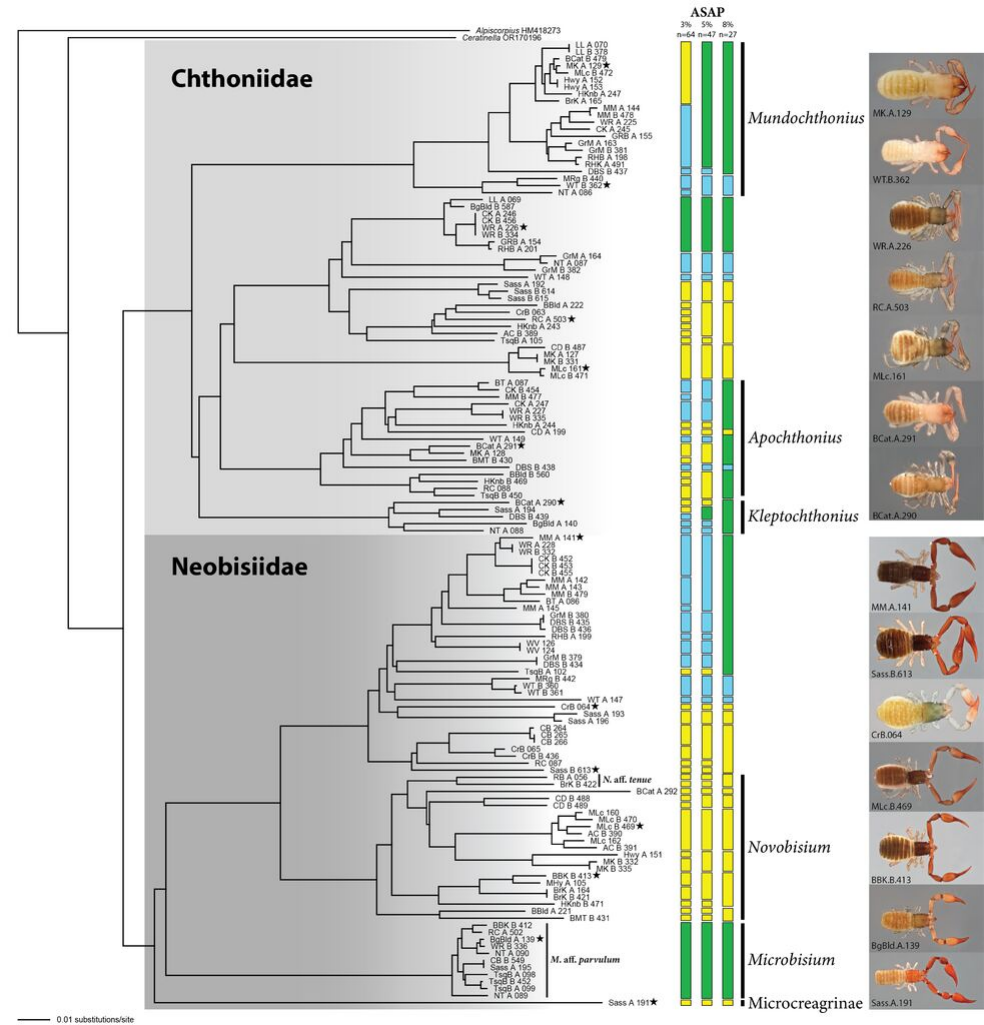
Neighbour-joining tree of Southern Appalachian Pseudoscorpiones barcoding sequences, based on K2P distances. Vertical boxes on the right of the tree indicated ASAP-inferred species level lineages, based on 3, 5 and 8% distance thresholds (yellow boxes: populations exclusively west of the Asheville Depression; blue boxes: populations to the east; green boxes: populations on both sides). Black stars indicate specimens illustrated on the right.

The barcodes obtained from our sampling are included in two main clades in both NJ and ML analyses (Fig. 2, Suppl. material 4), indicating that the pseudoscorpion fauna of the southern High Appalachian leaf litter is dominated by members of two families, Chthoniidae and Neobisiidae.

The best-scoring partition generated by ASAP analyses had little biological meaning, as delimited species were based on a distance threshold of only 0.35% and basically considering as single species only those individual specimens sharing a haplotype. For this reason, we present results from the next-best partitions, with more meaningful distance thresholds (Fig. 2, Suppl. material 1), covering the optimal identification thresholds observed in Chthoniidae (4.7% K2P nucleotide distance) and Neobisiidae (3.6%) (Hlebec et al. 2023b). With a less conservative threshold of 3% K2P genetic distance, ASAP estimates a total of 64 potential species. A higher interspecific threshold distance of 5% would estimate 47 species and an 8% threshold only 27 species.

Within the family Chthoniidae, ASAP estimated 33/22/12 species, depending on the distance threshold considered (3%/5%/8%). Two clades, including 11/7/6 species, remained identified only to the family level, but barcodes in three other clades could be assigned to genus, based on their phylogenetic placement. We found 5/3/2 species of *Mundochthonius* Chamberlin, 1929. *Apochthonius* Chamberlin, 1929 was represented by 17/12/5 species. *Kleptochthonius* Chamberlin, 1949 was represented by 5/4/1 species.

According to ASAP results, the Neobisiidae would include 31/25/15 species according to the 3%/5%/8% distance thresholds. About half of the barcodes could not be assigned to genus, representing 17/13/6 species, though one of them appears to correspond to the subfamily Microcreagrinae and others could represent different species of *Novobisium* Muchmore, 1967, but we are not certain about it. The phylogenetic position of several neobisiid haplotypes allowed placement, with a high certainty, in the genera *Novobisium*, with 13/10/7 species and *Microbisium* Chamberlin, 1930, with a single delimited species under all three thresholds, M.aff.parvulum (Banks, 1895).

Species diversity is slightly higher in the southern part of the region, which could be explained by a higher number of sampled localities in that area. Globally, ASAP analyses recover 39/26/15 species in localities west of the Asheville Depression biogeographic barrier, 23/17/6 east of it and 2/4/6 species with occurrences on both sides, but we observe differences amongst families, with diversity of Chthoniidae being more uniformly distributed than in Neobisiidae.

In the case of Chthoniidae, the ASAP results recover 18/9/4 species west of the Asheville Depression, 14/10/4 east of it and 1/3/4 on both sides. Thus, the pattern of higher diversity west of the Asheville Depression is observed only when delimiting species under the 3% distance threshold. Higher distance thresholds (5 and 8%) result in fewer species delimited by ASAP, with generally larger distributions and suggest the diversity on both sides of the Asheville Depression to be more similar.

Amongst Neobisiidae, diversity is higher in the south-western part of the region regardless of the delimitation parameters, with 21/17/11 species west of the Asheville Depression, 9/7/2 to the east and 1/1/2 on both sides. We observe a strong geographic structure in the clades related to *Novobisium*, with two of them present only west of the Asheville Depression and one almost exclusive to the eastern side. The only representative of *Microbisium* shows a wide distribution with only weak geographic structure and occurrences over most of the southern Appalachian Mountains.

## Discussion

The number of species estimated by the automated species delimitation method used here is largely dependent on the distance threshold considered. Our data including southern Appalachian barcodes do not show a clear barcoding gap marking the limit between intra- and interspecific distances. Analysing our 'whole GenBank dataset', we can find a weak barcoding gap ranging from 3 to 7% K2P distances, although with very little data on the intraspecific part of the histogram. These values agree with the optimal distance thresholds estimated for the families Chthoniidae and Neobisiidae in the Dinaric Alps in Europe (Hlebec et al. 2023b). Intraspecific genetic distances for some pseudoscorpion species have often been found to be relatively high, frequently around 8-10% uncorrected *p*-distances for *COI* sequences (e.g. Ohira et al. 2018a, Muster et al. 2021, Hlebec et al. 2023b). In most cases, observed high distances have been hypothesised (e.g Ohira et al. 2018b, Muster et al. 2021) or proved (e.g. Christophoryová et al. 2023, Hlebec et al. 2023a) to belong to undescribed, sometimes cryptic species (Hlebec et al. 2023b). In other arachnological studies focused mainly on spiders, intraspecific distances are usually well below 2.5% (Čandek and Kuntner 2014, Astrin et al. 2016) while most observed interspecific distances are higher than 8% (Barrett and Hebert 2005, Astrin et al. 2016). Considering all this information, all three considered thresholds are compatible with potentially valid species delimitation, although it is likely that the actual number of species is closer to the estimations, based on the 5-8% threshold. Those numbers differ little from the list by Muchmore and Cokendolpher of observed or expected species diversity in the Great Smoky Mountains for the families Chthoniidae (21 spp) and Neobisiidae (11 spp), although we do not think there is an exact correspondence of species amongst our delimited units and the available list of taxa. For instance, only single representatives of *Mundochthonius* or *Apochthonius* were reported by Lawson (1967) or listed by Muchmore and Cokendolpher (as *Mundochthonius* sp.), while our delimitation analyses suggest the existence in the region of 2 - 5 species of the former genus and 3 - 12 of the latter.

We must consider that our sampling is strictly focused on the leaf litter communities and more specifically to humid litter from high-elevation conifer forests. This should explain the absence of other families with known or expected presence in the Southern Appalachians. The families Chernetidae and Cheliferidae Risso, 1827 are likely represented by several genera and species in the region; however, it seems that they have different preferences, tending to occupy drier microhabitats, for instance, on dead or living trees under loosened bark (Muchmore 1973) and their presence in litter can be limited. Other species known from the region live in caves and are not expected to be found in litter although, given the right conditions, their presence in deep soil layers is also possible, as observed in other organisms (Jureková et al. 2021).

Most species delimited with low or moderate distance thresholds show a distribution restricted to single mountain ranges or at least geographically close ones. With the highest threshold, as expected, we observe more widespread units. Only two delimited species (Microvisiumaff.parvulum and a Chthoniidae lineage including samples LL.A.69, BgBld.B.587, CK.A.246, CK.B.456, WR.A.226, WR.B.334, GRB.A.154 and RHB.A.201) show a wide distribution regardless of the used threshold, with populations on both sides of the Asheville Depression, probably the most important biogeographic barrier in the Southern Appalachians (Caterino and Langton-Myers 2019 and references therein). Given their small size and terrestrial habits, the dispersal abilities of Pseudoscorpions are supposed to be limited. However, phoresy is a common phenomenon observed in this group. The use by non-flying organisms of flying species as vehicles for longer distance dispersals can result in increased distributional ranges and poorly-structured phylogeographic patterns (Poinar Jr et al. 1998, Opatova and Št'áhlavský 2018, Christophoryová et al. 2023, Sánchez‐Vialas et al. 2021), as is observed with our data for Microbisiumaff.parvulum. However, reports of phoresy in Chthoniidae and Neobisiidae are less frequent than in other families (Beier 1948, Poinar Jr et al. 1998) and it is still unknown if this is common behaviour in litter species.

There is a clear geographic pattern in Neobisiidae lineages related to *Novobisium*. Two groups of species are found only in the southern part of the region of study, west of the Asheville Depression, while in a third one, with just a few exceptions, species are restricted to the northeast of this pervasive barrier. This clear pattern suggests the operation of local speciation processes occurring during the last few millions of years, since this geographic feature is considered to have been an important barrier to dispersal since the late Miocene (Caterino and Langton-Myers 2018, Caterino and Langton-Myers 2019).

There is still much work to be done to fully characterise the diversity of Pseudoscorpiones in the southern Appalachian Mountains. The shortage of specialised taxonomists, aggravated by the death of William Muchmore in 2017 (Stephen and Harvey 2018), is the major drawback to advances in such an endeavour. Molecular methods like DNA barcoding, applied here for the first time to the pseudoscorpion fauna of this region, indeed give us an idea of the patterns of diversity associated with leaf litter communities (e.g., Dukes et al. 2022, Caterino and Recuero 2023a, Caterino and Recuero 2023b). The next step must be to integrate these molecular data with morphological evidence, to provide more solid species delimitations (e.g. Caterino 2022, Recuero and Caterino 2023) that will guide future efforts on the study of the evolutionary history, ecology and conservation of these minute, but important members of the soil and litter faunas.

## Supplementary Material

E1107EFB-7FAD-5FC2-85EE-4C35E23E8D44 10.3897/BDJ.12.e115928.suppl1 10499949 Supplementary material 1 Collecting and voucher information for Pseudoscorpiones barcode sequences Data type occurrence Brief description An Excel spreadsheet containing specimen collecting data (locality, date, lat/long), voucher codes, DNA extraction codes and GenBank accession numbers for all sequences reported. File: oo_962105.xlsx https://binary.pensoft.net/file/962105 Recuero, E. & Caterino, M. S.

99E550CD-19D3-5B9D-BC0A-F956B6735C2C 10.3897/BDJ.12.e115928.suppl2 10499951 Supplementary material 2 Cytochrome Oxidase I barcode region sequences for Appalachian Pseudoscorpiones Data type phylogenetic Brief description 137 partical COI sequences of Pseudoscorpiones from the southern Appalachians, in nexus format. File: oo_936443.nex https://binary.pensoft.net/file/936443 Recuero, E. & Caterino, M. S.

BEAAC03F-0767-53EF-B4BF-46D628E6A243 10.3897/BDJ.12.e115928.suppl3 Supplementary material 3 GenBank sequences used for ML analysis Data type Table in XLSX format Brief description An excel spreadsheet containing the GenBank sequences used for ML analysis, including accession numbers, taxonomic information and references. File: oo_958567.xlsx https://binary.pensoft.net/file/958567 Recuero, E. & Caterino, M. S.

DCFDAB3D-C7FE-509D-8C9A-8F7E7030B06D 10.3897/BDJ.12.e115928.suppl4 Supplementary material 4 Maximum Likelihood tree including southern Appalachian and GenBank barcodes of Pseudoscorpiones Data type Phylogenetic tree in nexus format Brief description Maximum Likelihood tree obtained from southern Appalachian and GenBank barcodes of Pseudoscorpiones, generated using IQtree. Node support is measured with ultrafast bootstrap. File: oo_958566.nexus https://binary.pensoft.net/file/958566 Recuero, E & Caterino, M. S.
